# Pulmonary Metastatic Calcifications Secondary to Chronic Renal Failure

**DOI:** 10.7759/cureus.84862

**Published:** 2025-05-26

**Authors:** Chandrashekar Patil, Polneni Lavanya, Nikhitha Mangalagiri, Deepa Bommanagari, Paras Gupta

**Affiliations:** 1 Radiology, Mallareddy Medical College for Women, Hyderabad, IND; 2 Radiodiagnosis, Mallareddy Medical College for Women, Hyderabad, IND

**Keywords:** alveolar microliths, calcium, chronic renal failure, metastatic calcification, multiple centrilobular calcified nodules

## Abstract

Pulmonary metastatic calcification (PMC) is a rare but significant condition associated with chronic renal failure, particularly in patients undergoing long-term hemodialysis. PMC results from abnormal calcium-phosphate metabolism, leading to ectopic deposition of calcium salts in lung tissues. It can also occur due to hypercalcemia, primary and secondary hyperparathyroidism, excessive exogenous administration of calcium and vitamin D, sarcoidosis, milk-alkali syndrome, osteoporosis, renal or liver transplantation, and Paget’s disease. Pathogenesis involves hyperphosphatemia, secondary hyperparathyroidism, and systemic dysregulation of calcium homeostasis. This condition is often asymptomatic but may present with progressive dyspnea, hypoxemia, or radiographic abnormalities such as diffuse or nodular opacities on imaging studies. Diagnosis relies on a combination of clinical history, laboratory findings, imaging, and, in some cases, histological confirmation. Management focuses on correcting the underlying metabolic derangements, including controlling serum phosphate and calcium levels through dietary restrictions, phosphate binders, calcimimetics, and optimizing dialysis. In severe cases, lung transplantation may be considered. Early recognition and prompt intervention are crucial to prevent complications and enhance patient outcomes. Here, we present a 60-year-old patient with pulmonary metastatic calcifications secondary to chronic renal failure.

## Introduction

Pulmonary metastatic calcification (PMC) is a rare condition characterized by abnormal calcium deposition in lung tissue, most commonly observed in patients with chronic renal failure (CRF), particularly those with ESRD (end-stage renal disease) on hemodialysis. Metastatic calcification of the lung is linked to various complications, including impaired gas exchange, pulmonary fibrosis, and, in some cases, even death [1]. This condition arises due to disruptions in calcium and phosphate metabolism, often associated with secondary hyperparathyroidism and hyperphosphatemia, which are common in advanced renal disease. PMC primarily affects the alveolar septa, bronchial walls, and blood vessels. Although often asymptomatic and discovered incidentally on imaging, PMC can occasionally lead to respiratory symptoms, such as dyspnea and hypoxemia, as calcification progresses. Radiographic findings, including diffuse, patchy, or nodular opacities, can resemble other pulmonary conditions, complicating diagnosis. Histological examination is considered the gold standard for confirmation but is rarely performed due to associated risks. Given the rising prevalence of chronic kidney disease and dialysis dependence, the incidence of PMC is expected to increase. Despite its potential to impact respiratory function and quality of life, PMC remains under-recognized in clinical practice.

## Case presentation

**Figure 1:**
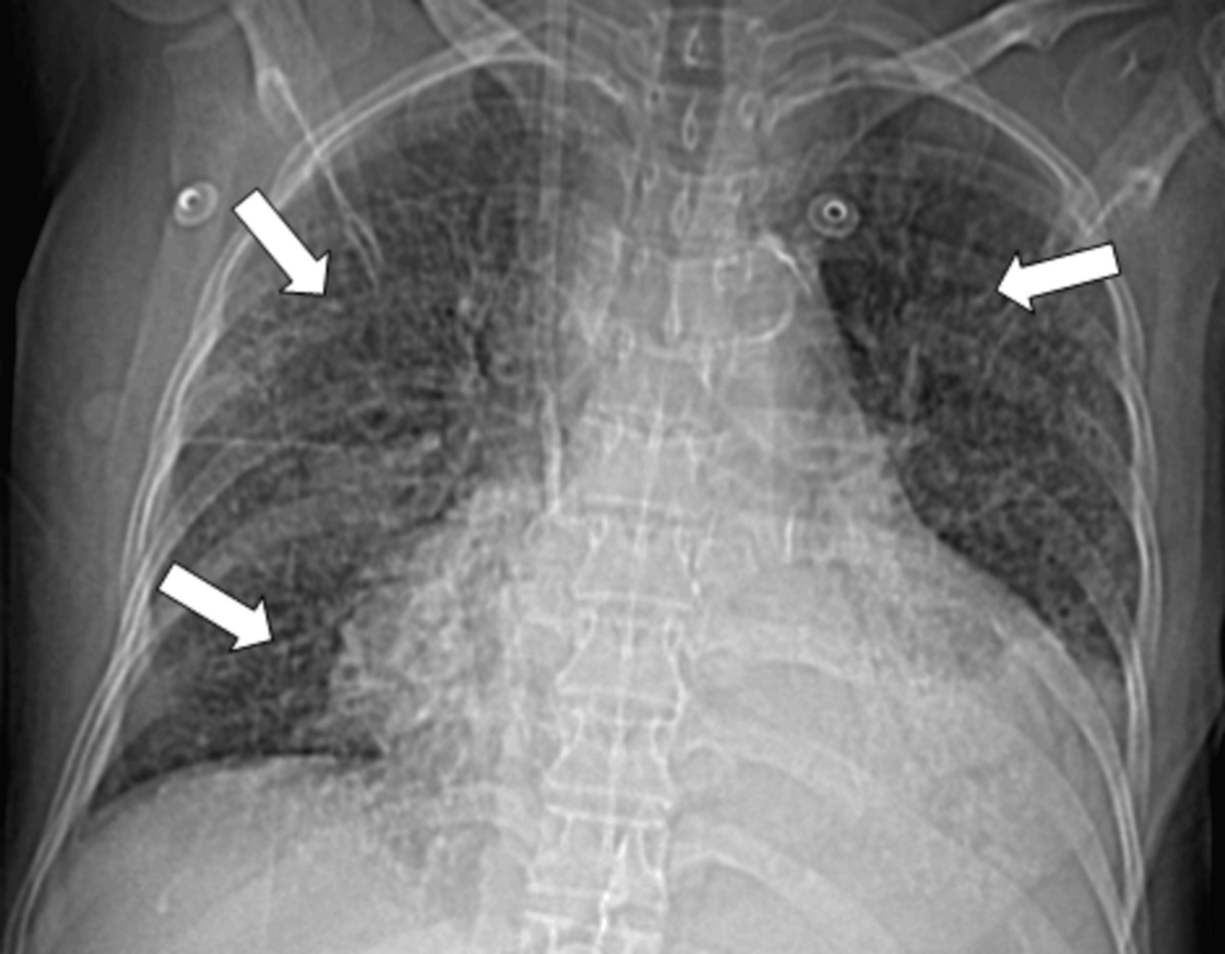
Chest X-ray AP view shows multiple tiny hyperdense foci in bilateral lung fields (indicated by arrows). AP: Anteroposterior

**Figure 2:**
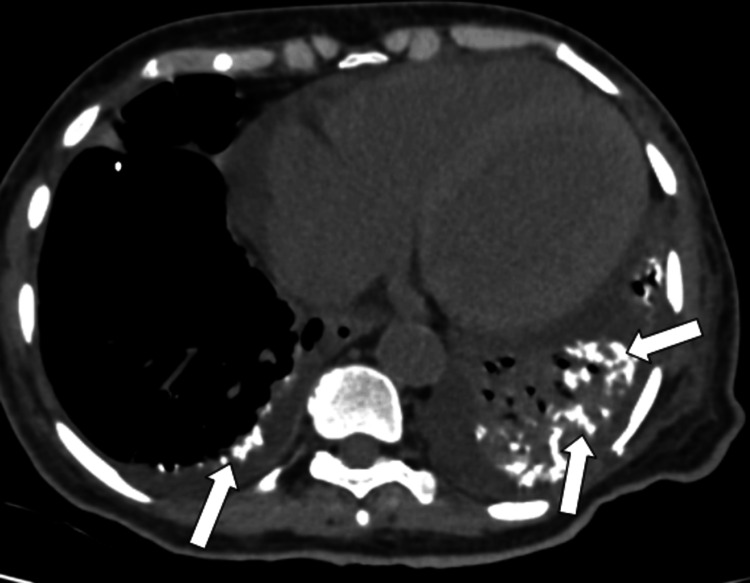
CT axial sections, soft tissue window plain images showing multiple patchy peribronchial calcifications in the left lower lobe and some calcifications along the right lower lobe pleura (indicated by arrows). CT: Computed tomography

**Figure 3:**
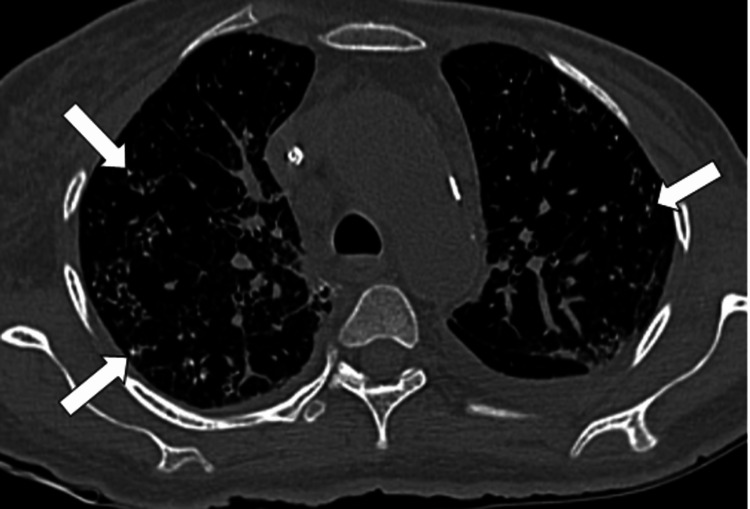
Axial CT chest plain lung window: Note multiple tiny calcifications diffusely scattered in bilateral lungs (indicated by arrows). CT: Computed tomography

**Figure 4:**
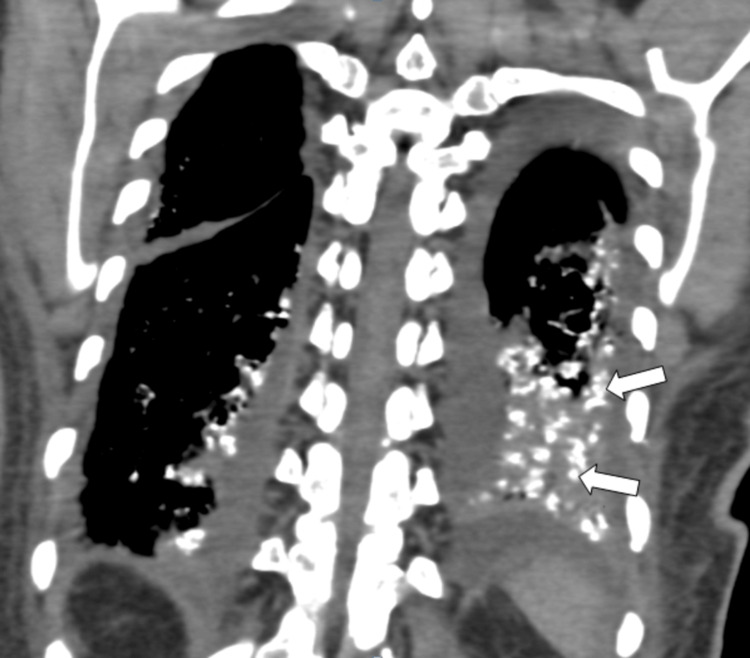
CT coronal image of the same patient showing left lobar peribronchial calcifications (indicated by arrows). CT: Computed tomography

**Figure 5:**
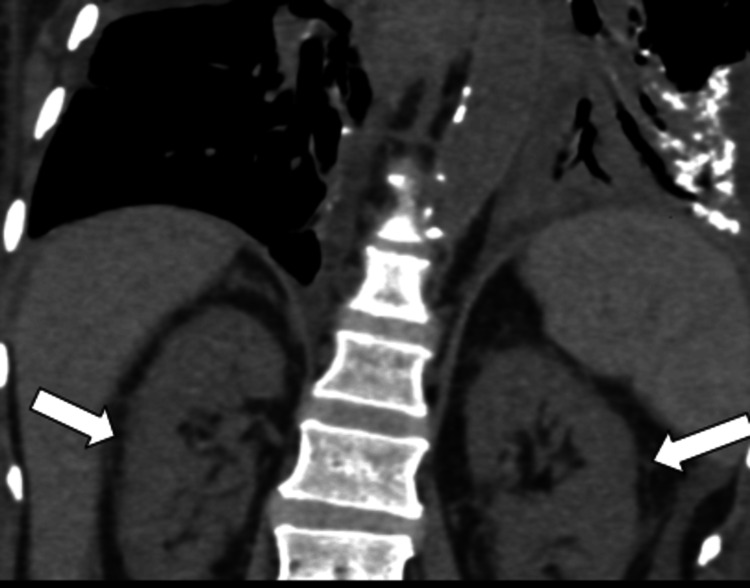
Coronal image of the same patient, showing bilateral mild perinephric fat stranding.

A 60-year-old female came to the outpatient clinic with complaints of progressive shortness of breath on exertion, pedal edema, fever, and difficulty in swallowing and was admitted to our center. The patient had no history of orthopnea or paroxysmal nocturnal dyspnea. She has been a known case of chronic kidney disease due to uncontrolled diabetes for 8 years and is on regular hemodialysis. Auscultation of the lungs revealed inspiratory coarse crackles, occasional wheezing, and diminished vesicular breath sounds throughout all thoracic areas, especially at the bilateral lung bases. Cardiac auscultation was unremarkable. The rest of her systemic examination was normal. Sputum cultures were negative. She had no history of dust exposure or cardiac problems. Her blood oxygen saturation was 96% with 2 liters of oxygen. The patient had a history of percutaneous transluminal coronary angioplasty. Her laboratory test results are mentioned (Table 1). Chest X-ray shows multiple tiny hyperdense foci in bilateral lung fields (Figure 1), which were better appreciated on CT. HRCT chest images demonstrated (Figures 2-4) multiple peribronchial tiny irregular calcific micronodules seen extensively scattered in bilateral lungs, more in bilateral lower lobes (left > right)-s/o metastatic calcifications, mild diffuse bronchiectasis, subtle diffuse smooth septal thickening seen in bilateral lungs, patchy pleural calcifications seen in bilateral lower lobes, left mild pleural effusions with collapse consolidation of underlying lung segments, and loculated right pleural effusion. Bilateral mild perinephric fat stranding (Figure 5).

**Table 1:** A list of laboratory values on admission. BUN: Blood urea nitrogen

Test	Result	Normal Range
Total Leukocyte Count (TLC)	6500/cmm	4000-11000/cmm
Hemoglobin	8.5 g/dl	12-16 g/dl (women)
Neutrophils	75%	40-75%
Lymphocytes	12%	20-40%
Liver Function Tests	Normal	Normal
Serum Parathyroid Hormone	40 pg/mL	10- 65 pg/mL
Serum Electrolytes	Normal	Normal
Vitamin D	22 ng/mL	20-29 ng/mL
BUN	16 mg/dl	7.0-25.0 mg/dl
Serum Creatinine	3.2 mg/dl	0.3-1.4 mg/dl
Calcium	15.8 mg/dl	8.4-10.2 mg/dl
Phosphorus	3.8 mg/dl	2.7-4.5 mg/dl

A diagnosis of pulmonary metastatic calcifications secondary to chronic renal failure was made. The patient was treated with Magnex Forte (a combination of cefoperazone [1g] and sulbactam [500mg]), clopidogrel (an antiplatelet), Meaxon Plus (a combination of methylcobalamin [1 mg] and alpha lipoic acid [100 mg]), vitamin D, bisphosphonates, and other supplements and is doing well now with resolution of shortness of breath (SOB), pedal edema, and normal calcium levels with regular follow-up.

## Discussion

Metastatic calcification involves the deposition of calcium in normal, non-damaged tissue. The lungs are a common site for this type of calcium accumulation [2]. Pulmonary metastatic calcifications are typically associated with persistently elevated serum calcium-phosphorus product, as seen in conditions such as chronic renal failure, primary hyperparathyroidism, vitamin D hypervitaminosis, milk-alkali syndrome, or diffuse myelomatosis [3].

Calcium salts are primarily deposited in the alveolar walls, with lesser deposition in the bronchial walls, pulmonary arteries, and veins [4]. Calcium tends to accumulate in tissues with relatively alkaline environments, which explains why the lung apices are more frequently affected than the lung bases. Interestingly, the severity of respiratory distress does not always correspond to the extent of calcification. Patients with significant calcification may remain asymptomatic, while others with minimal calcification or normal chest radiographs can experience severe respiratory compromise [5].

Metastatic pulmonary calcification (MPC) is a well-recognized complication of end-stage renal failure, often identified at autopsy, though many patients remain asymptomatic. When symptoms do occur, they include dyspnea and a chronic, non-productive cough. Chest radiographs are not highly effective in detecting small calcifications, often showing normal results or mimicking conditions like pulmonary edema or pneumonia. High-resolution CT (HRCT) is more sensitive, revealing various patterns such as diffuse calcified nodules, ground-glass opacities, or lobar consolidation, which can mimic pneumonia. MPC can present in various patterns, including as a diffuse interstitial process or as discrete and confluent calcified nodules [6]. Radionuclide imaging using technetium-99m is also useful in early detection, identifying calcification before it appears on chest X-rays.

MPC can be mistaken for calcifications due to infections (e.g., tuberculosis or histoplasmosis), alveolar microlithiasis, silicosis, or metastatic malignancies. However, its characteristic patterns in the upper lung zones, along with calcification in the myocardium, bronchial walls, and chest wall vessels, make it most likely in patients with chronic renal failure. 

HRCT remains a crucial diagnostic tool, often replacing the need for invasive procedures like lung biopsies. Recognizing the distinct HRCT patterns associated with MPC is essential for timely diagnosis and appropriate management.

Pulmonary calcification associated with renal failure can be potentially reversible and may improve with treatments such as parathyroidectomy, renal transplantation, or proper dialysis. In certain cases, symptom resolution has been observed following the correction of hypercalcemia.

Ozlem Alkan et al. presented a 47-year-old patient with chronic renal failure, undergoing hemodialysis for 10 years, who presented with persistent shortness of breath for three months. An HRCT was performed, revealing multiple centrilobular calcified nodules and patchy areas of ground-glass opacity throughout both lungs. The findings showed a symmetrical distribution affecting both lungs. Additionally, there was a thin rim of calcification that encircled the trachea and main bronchi. The patient underwent subtotal parathyroidectomy and is doing well [7].

## Conclusions

Pulmonary metastatic calcification (PMC) is a frequently underdiagnosed condition, especially in patients with ESRD undergoing dialysis, and while it often remains asymptomatic, it can lead to severe complications such as irreversible lung damage and respiratory failure if left untreated. HRCT is a critical diagnostic tool, providing a non-invasive, highly sensitive method for detecting pulmonary calcifications, which are often seen as nodules or masses in the lungs. Unlike open lung biopsy, which carries risks such as infection and bleeding, HRCT offers clear, detailed imaging that helps confirm the diagnosis and eliminate the need for invasive procedures. Clinicians must maintain a high index of suspicion when dialysis patients present with unexplained respiratory symptoms such as dyspnea, cough, or chest pain, especially given the common metabolic disturbances in ESRD, including hyperphosphatemia and hypercalcemia, that predispose them to PMC. Early recognition and intervention are essential to correct calcium-phosphate imbalances, reduce progression, and prevent irreversible lung damage, ultimately improving patient outcomes.
